# Situación actual de las enfermedades huérfanas en Bogotá: notificación al Sivigila entre el año 2019 y 2022

**DOI:** 10.15446/rsap.V25n4.107594

**Published:** 2023-07-01

**Authors:** Julián Serrano-Giraldo, Martha P. Becerra-Muñoz, Jennifer A. Tijaro-Santos, Ignacio Zarante

**Affiliations:** 1 JS: MD. Facultad de Medicina, Pontificia Universidad Javeriana. Bogotá, Colombia serrano.julian@javeriana.edu.co Pontificia Universidad Javeriana Facultad de Medicina Pontificia Universidad Javeriana Bogotá Colombia serrano.julian@javeriana.edu.co; 2 MB: MD. M. Sc. Salud Pública. Secretaría de Salud. Bogotá, Colombia. mpbecerra@saludcapital.gov.co Salud Pública Secretaría de Salud Bogotá Colombia mpbecerra@saludcapital.gov.co; 3 JT: FGA. Secretaría de Salud. Bogotá, Colombia. jatijaro@saludcapital.gov.co Secretaría de Salud Bogotá Colombia jatijaro@saludcapital.gov.co; 4 IZ: MD. M. Sc. Ciencias Biológicas. Ph. D. Ciencias. Instituto de Genética Humana, Pontificia Universidad Javeriana. Bogotá, Colombia. izarante@javeriana.edu.co Pontificia Universidad Javeriana Instituto de Genética Humana Pontificia Universidad Javeriana Bogotá Colombia izarante@javeriana.edu.co

**Keywords:** Enfermedades raras, monitoreo epidemiológico, salud pública, tiempo hasta el diagnóstico, retraso diagnóstico (fuente: DeCS, BIREME), Rare diseases, epidemiological monitoring, public health, time to diagnosis, diagnostic delay (source: MeSH, NLM)

## Abstract

**Objetivo:**

Analizar los reportes de enfermedades huérfanas en Bogotá, con el fin de describir el perfil epidemiológico, a partir de los casos notificados al Sistema de Salud Pública (Sivigila), de enero de 2019 hasta marzo de 2022.

**Métodos:**

Se realizó un estudio descriptivo y transversal en el que se analizaron los casos notificados al Sistema Nacional de Vigilancia en Salud Pública (Sivigila) en Bogotá en el periodo comprendido de enero de 2019 y marzo de 2022. Se calcularon frecuencias absolutas y relativas, distribución de frecuencia y prevalencias y promedios de distintas variables notificadas en las fichas de notificación.

**Resultados:**

Desde enero de 2019 hasta marzo de 2022 se han notificado al Sivigila en Bogotá 10 399 pacientes con enfermedades huérfanas, de los cuales el 56,25% (5 849) corresponde al sexo femenino y el 43,75% (4 550) al sexo masculino. El 87,10% (9 060) de los casos pertenece al régimen contributivo. La localidad con mayor cantidad de reportes fue Suba con el 15,85% (1 294). Las enfermedades huérfanas más notificadas fueron: la esclerosis múltiple con el 13,1% (1 363), la esclerosis lateral amiotrófica con el 4,04% (421) y el síndrome de Guillain-Barre con el 3,6% (374). Un paciente con una enfermedad huérfana en Bogotá tarda desde que inician sus síntomas hasta obtener un diagnóstico 61,3 meses en promedio (DE 101,9).

**Conclusiones:**

A partir de la notificación al Sivigila en Bogotá, en comparación con la prevalencia mundial, hay un subregistro de los pacientes con enfermedades huérfanas y el retraso en el diagnóstico de estas enfermedades es evidente.

## Abstract (en)

**Objective:**

To analyze the reports of orphan diseases in Bogotá, in order to describe the epidemiological profile, based on the cases reported to the Public Health System (Sivigila), from January 2019 to March 2022.

**Methods:**

A descriptive and cross-sectional study was carried out in which the cases reported to Sivigila in Bogotá were analyzed in the period between January 2019 and March 2022. Absolute and relative frequencies, frequency distribution and prevalences and averages of different variables were calculated. notified in the notification sheets.

**Results:**

From January 2019 to March 2022, 10,399 patients with orphan diseases have been notified to Sivigila in Bogotá, of which 56.25% (5,849) are female and 43.75% (4,550) are female. male sex. 87.10% (9,060) of the cases belong to the contributory regime. The town with the highest number of reports was Suba with 15.85% (1,294). The most reported orphan diseases were: multiple sclerosis with 13.1% (1,363), amyotrophic lateral sclerosis with 4.04% (421) and Guillain-Barre syndrome with 3.6% (374). A patient with an orphan disease in Bogotá takes 61.3 months on average from the beginning of their symptoms to obtaining a diagnosis (SD 101.9).

**Conclusions:**

From the notification to Sivigila in Bogotá, compared to the global prevalence, there is an under-registration of patients with orphan diseases and the delay in the diagnosis of these diseases is evident.

El término enfermedades raras se usó por primera vez en Estados Unidos a mediados de los años ochenta 1. La expresión enfermedades huérfanas se usó inicialmente para denotar a las patologías desatendidas, es decir, en las cuales la investigación sobre su diagnóstico y tratamiento es escasa. Sin embargo, hay algunas enfermedades comunes que podrían considerarse huérfanas, porque afectan sobre todo a países de bajos ingresos y suelen haber pocos incentivos financieros para investigarlas 2.

El 1.o de diciembre de 1999 se aprobó el primer Plan de Acción Comunitaria sobre las enfermedades poco comunes, el cual estableció como definición de enfermedad rara a las patologías cuya prevalencia esté por debajo de cinco casos por cada 10 000 personas. No obstante, esa definición solo fue válida para la Unión Europea, actualmente para ellos enfermedad rara es aquella que tenga una prevalencia menor a 1 por cada 2 000. En Estados Unidos se define una enfermedad como rara si solo afecta a menos de 200 000 personas en todo el país, lo que es equivalente a 1: 1 600 personas. En Japón enfermedad rara es la que afecta a menos de 50 000 personas en su país o que tenga una prevalencia de 1 por cada 2 500 1,2.

En Argentina, dado el estigma que evocan expresiones como "raras" o "huérfanas", han adoptado el término de enfermedades poco frecuentes y las definen como "aquellas cuya prevalencia poblacional es igual o inferior a 1 en 2 000 personas, referida a la situación epidemiológica nacional" 3.

De forma reciente, algunos autores han ampliado esta definición, como en Colombia, donde el Ministerio de Salud y Protección Social define una enfermedad huérfana o rara en la Ley 1392 de 2010 como "aquella crónicamente debilitante, grave, que amenazan la vida y con una prevalencia menor de 1 por cada 5 000 personas, comprenden, las enfermedades raras, las ultra-huérfanas y olvidadas" 4. En el mundo se han identificado entre 6 000 y 8 000 enfermedades huérfanas, de las cuales Colombia ha incluido 2 247 en la Resolución 023 del 2023 2,5.

Las enfermedades huérfanas como grupo son comunes. Se estima que entre el 6% y el 8% de la población mundial padece una enfermedad huérfana, lo que se traduce entre 470 y 620 millones de personas 6, y extrapolado a Colombia serían 3 a 4 millones de afectados. La mayoría de ellas son catalogadas como enfermedades de origen genético, as las que se suman algunos tipos raros de cáncer, trastornos autoinmunes, malformaciones congénitas, entre otras. Por lo general, suelen ser enfermedades altamente debilitantes, crónicas, con múltiples deficiencias motoras, sensoriales y cognitivas; por lo tanto, suelen presentar un alto nivel de complejidad clínica que dificulta su reconocimiento y diagnóstico 1.

El desconocimiento por parte del personal de salud, en especial en hospitales de baja y mediana complejidad, de muchas enfermedades huérfanas lleva a retrasos en el diagnóstico y remisión hacia centros de referencia. Los retrasos en el diagnóstico pueden llevar a progresión de la enfermedad, deterioro de la calidad de vida, discapacidad, tratamientos farmacológicos y no farmacológicos innecesarios e incluso a una mayor mortalidad. Por ende, se puede impactar de manera positiva en la historia natural de la enfermedad mediante el desarrollo de estrategias que permitan capacitar al personal de salud en el reconocimiento temprano de enfermedades huérfanas 1,7; así mismo, si se avanza hacia un enfoque de sensibilización, prevención, vigilancia y control de estas patologías se obtendría un gran beneficio para el sistema de salud, dado que la atención de las enfermedades huérfanas representa un costo económico elevado por la prolongada atención sanitaria especializada que requieren y los medicamentos, muchos de alto costo, que se requieren en su manejo 8.

Bogotá, capital de Colombia, se encuentra a una altura de 2 582 metros sobre el nivel del mar. Es la ciudad más poblada del país, con 7 901 653 habitantes para 2022. Se encuentra conformada por 20 localidades, 19 urbanas y una rural, Sumapaz. La localidad más poblada para 2022 es Suba, con 1 273 909 habitantes, en tanto que Sumapaz es la menos poblada, con 3 713 habitantes. La localidad con la mayor densidad poblacional para el 2022 fue Bosa, con 30 131 habitantes/km2, y Sumapaz fue la menos densa, con 4,75 habitantes/km2 9,10.

Con base en lo anterior, se decidió elaborar el perfil epidemiológico de las personas con enfermedades huérfanas notificadas al Sistema de Vigilancia en Salud Pública (Sivigila) de Bogotá, para el periodo comprendido entre enero de 2019 y marzo de 2022, mediante el análisis de los variables sociodemográficas.

## MATERIALES Y MÉTODOS

Se llevó a cabo un estudio descriptivo y transversal de los casos notificados al Sistema Nacional de Vigilancia Epidemiológica (Sivigila) en el periodo comprendido entre el 1.o de enero de 2019 y el 31 de marzo de 2022 del evento de reporte obligatorio de enfermedades raras "342" 11. Se analizaron las variables edad, sexo, tipo de régimen de salud, tipo de documento, localidad de residencia del caso. Se excluyeron los reportes repetidos e incompletos que no diligenciaron las variables de interés.

Para las variables demográficas se hizo una distribución de frecuencias usando Microsoft Excel (versión 18.0). Se calculó la prevalencia y las frecuencias absolutas y relativas de la ocurrencia de las enfermedades huérfanas de cada localidad de Bogotá, en tanto que la distribución geográfica de la prevalencia por localidad se mapeó con el programa QGis (versión 3.26.0).

La prevalencia se definió como el número de casos dividido sobre el total de la proyección de la población del grupo en cuestión, multiplicado por un coeficiente de 10 000 habitantes. Los datos demográficos para el cálculo de las prevalencias se obtuvieron de las proyecciones de las localidades de Bogotá para el periodo 2018-2035 10 del Departamento Administrativo Nacional de Estadística (DANE).

Se obtuvieron las 10 enfermedades huérfanas más frecuentes reportadas desde el 1.° enero de 2019 hasta el 31 de marzo de 2022, y luego las 5 más frecuentes de acuerdo con año y grupo de edad; a cada tabla obtenida se le realizó una distribución de frecuencia con la variable sexo, y se calculó la prevalencia con la misma metodología descrita a partir de los datos demográficos obtenidos de las proyecciones de población a nivel departamental, periodo 2018-2050 del DANE 10 y del Observatorio de Salud de Bogotá (SaluData) 12, multiplicado por coeficiente de 100 000 habitantes.

Se calculó el tiempo promedio en meses, la mediana y la desviación estándar del tiempo de la diferencia entre el inicio de los síntomas y la fecha de confirmación diagnóstica de las 5 enfermedades huérfanas más frecuentes en función de la demora diagnóstica.

## RESULTADOS

**Figura 1:**
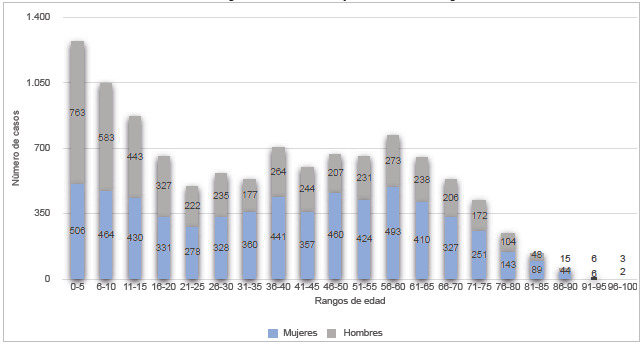
Distribución por grupos de edades y sexo de casos de enfermedades huérfanas notificadas al Sivigila entre enero de 2019 y marzo de 2022 en Bogotá D. C.

Entre el 1.o enero de 2019 y el 31 de marzo de 2022, se notificaron al Sivigila 10 399 personas con enfermedades huérfanas en Bogotá, de las cuales 5 840 (56,25%) corresponden al sexo femenino y 4 550 (43,75%) al sexo masculino. Las características de la población se muestran en la Tabla 1. La mayoría de los casos corresponde al grupo etario de los 25 a los 60 años (4 384, 42,15%); el grupo de edad con la mayor cantidad de casos fue de los 0 a los 5 años, con 1 214 (11,67%) reportes, y cabe resaltar que aquí el 60,3% (732) de dichos casos corresponde al sexo masculino (Figura 1). La gran mayoría de los casos pertenece al régimen contributivo (9 060, 87,1%).

**Tabla 1:** Características sociodemográficas de casos de enfermedades huérfanas notificadas al Sivigila entre enero de 2019 y marzo de 2022 en Bogotá D. C.

Variable	Categoría	2019	2020	2021	2022*	Total
n (%)	n (%)	n (%)	n (%)	n (%)
Sexo	Femenino	2 753 (58,4)	1 536 (56,12)	1 367 (52,58)	193 (55,46)	5 849 (56,25)
	Masculino	1961 (41,6)	1201 (43,8)	1233 (47,42)	155 (44,54)	4550 (43,75)
Grupo Etario	0-5	435 (9,22)	314 (11,47)	408 (15,69)	57 (16,38)	1214 (11,67)
	6-12	578 (12,25)	365 (13,34)	355 (13,65)	40 (11,49)	1338 (12,86)
	13-18	427 (9,05)	208 (7,6)	246 (9,46)	25 (7,18)	906 (8,71)
	19-24	242 (5,13)	166 (6,07)	149 (5,73)	15 (4,63)	572 (5,5)
	25-60	2153 (45,6)	1129 (41,25)	970 (37,31)	131 (37,64)	4384 (42,15)
	>60	879 (18,68)	555 (20,28)	472 (18,15)	80 (22,61)	1988 (19,11)
Tipo de Régimen	Contributivo	4324 (91,71)	2106 (76,95)	2311 (88,88)	317 (91,09)	9060 (87,10)
	Subsidiado	279 (5,91)	158 (5,77)	242 (9,31)	24 (6,9)	703 (6,76)
	Indeterminado/Pendiente	51 (1,08)	422 (15,42)	11 (0,42)	5 (1,44)	489 (4,7)
	Especial	53 (1,14)	21 (0,77)	0	0	75 (0,72)
	Excepción	5 (0,11)	11 (0,40)	20 (0,77)	0	36 (0,35)
	No asegurado	2 (0,04)	19 (0,69)	16 (0,62)	2 (0,54)	39 (0,37)
Tipo de Documento	Cedula de ciudadanía	3292 (69,85)	1868 (68,25)	1592 (61,23)	220 (63,22)	6975 (67,05)
	Registro civil de nacimiento	658 (13,95)	410 (14,98)	508 (19,54)	72 (20,69)	1648 (15,84)
	Tarjeta de Identidad	702 (14,88)	379 (13,85)	393 (15,12)	42 (12,07)	1516 (14,57)
	Menor sin Identificar	23 (0,49)	57 (2,08)	42 (1,62)	3 (0,86)	125 (1,16)
	Cedula de extranjería	35 (0,74)	7 (0,26)	16 (0,62)	2 (0,57)	60 (0,58)
	Certificado de nacido vivo	0	6 (0,22)	34 (1,31)	6 (1,72)	46 (0,44)
	Permiso especial de permanencia	2 (0,04)	6 (0,22)	9 (0,35)	2 (0,57)	19 (0,18)
	Pasaporte	2 (0,04)	4 (0,15)	2 (0,08)	0	8 (0,08)
	Salvoconducto	0	0	3 (0,12)	0	3 (0,03)
	Adulto sin identificar	0	0	0	1 (0,29)	1 (0,01)
	Documento extranjero	0	0	1 (0,04)	0	1 (0,01)
Total		4 714 (100)	2 737 (100)	2 600 (100)	348 (100)	10 399 (100)

**Figura 2:**
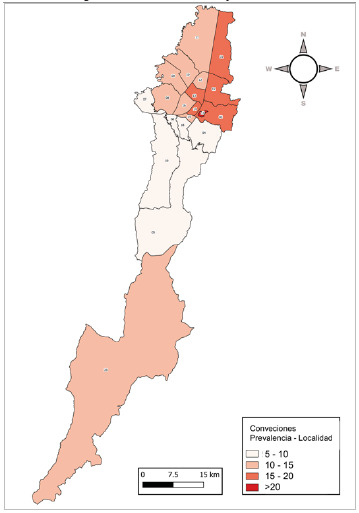
Prevalencia de enfermedades huérfanas por localidades de Bogotá D.C, entre enero de 2019 y marzo de 2022

Para el periodo estudiado y analizado, la localidad de residencia que concentra la mayoría de los reportes es Suba, con 1 294 casos (15,85%). La localidad con la mayor prevalencia fue La Candelaria (34,07 por cada 10 000 habitantes). Para al análisis de la prevalencia por localidades se usaron 8 162 reportes. La Tabla 2 y la Figura 2 muestran la distribución de la prevalencia por localidades.

**Tabla 2:** Prevalencia de enfermedades huérfanas por localidades de Bogotá D.C, 2019 -marzo de 2022

Localidad	2019		2020		2021		2022*	Total	
	n (%)	Prevalencia+	n (%)	Prevalencia+	n (%)	Prevalencia+	n (%)	n (%)	Prevalencia
11. Suba	580 (16,32)	4,86	368 (16,92)	3,00	294 (13,71)	2,35	52 (18,06)	1294 (15,85)	10,54
08. Kennedy	409 (11,51)	3,98	286 (13,15)	2,76	288 (13,43)	2,78	36 (12,5)	1019 (12,48)	9,85
10. Engativá	447 (12,58)	5,57	267 (12,28)	3,29	245 (11,42)	3,01	37 (12,85)	996 (12,20)	12,27
01. Usaquén	418(11,76)	7,59	212 (9,74)	3,76	174 (8,11)	3,05	27 (9,38)	831 (10,18)	14,72
07. Bosa	246 (6,92)	3,48	145 (6,67)	2,02	168 (7,83)	2,3 2	21 (7,29)	580 (7,11)	8,08
19. Ciudad Bolívar	167 (4,7)	2,65	91 (4,18)	1,42	129 (6,01)	1,99	25 (8,68)	412 (5,05)	6,43
09. Fontibón	172 (4,84)	4,56	106 (4,87)	2,74	101 (4,71)	2,56	13 (4,51)	392 (4,80)	10,13
04. San Cristóbal	118 (3,32)	3,01	78 (3,59)	1,96	121 (5,6 4)	3,02	9 (3,13)	326 (3,99)	8,21
18. Rafael Uribe Uribe	129 (3,63)	3,46	81 (3,72)	2,13	102 (4,76)	2,66	10 (3,47)	322 (3,92)	8,47
16. Puente Aranda	138 (3,88)	5,58	72 (3,31)	2,87	83 (3,87)	3,27	9 (3,13)	302 (3,70)	12,03
02. Chapinero	152 (4,28)	9,31	71 (3,26)	4,18	69 (3,22)	3,98	9 (3,13)	301 (3,59)	17,73
13. Teusaquillo	144(4,05)	9,44	66 (3,03)	4,09	72 (3,36)	4,29	4 (1,39)	286 (3,50)	17,74
05. Usme	95(2,67)	2,53	83 (3,82)	2,15	82 (3,82)	2,08	14 (4,86)	274 (3,36)	7,12
12. Barrios Unidos	89 (2,50)	6,43	58 (2,67)	4,05	44 (2,05)	3,00	3 (1,04)	194 (2,38)	13,54
03. Santa Fe	75 (2,11)	7,08	50 (2,30)	4,65	48 (2,24)	4,45	8 (2,78)	181 (2,22)	16,84
06. Tunjuelito	58 (1,63)	3,30	49 (2,25)	2,74	42 (1,96)	2,33	3 (1,04)	152 (1,86)	8,50
14. Los Mártires	52 (1,46)	6,27	45 (2,07)	5,38	34 (1,59)	4,08	3 (1,04)	134 (1,64)	16,03
15. Antonio Nariño	39 (1,10)	4,87	23 (1,06)	2,83	37 (1,72)	4,50	3 (1,04)	102 (1,25)	12,52
17. La Candelaria	24 (0,68)	13,83	23 (1,06)	13,06	11 (0, 51)	6,15	2 (0,69)	60 (0,72)	34,07
20. Sumapaz	2 (0,06)	6,06	1 (0,05)	2,90	1 (0,04)	2,79	0	4 (0,05)	11,60
Total	3554 (100)		2175 (100)		2145 (100)		268 (100)	8162 (100)	-

Prevalencia multiplicada por coeficiente de 10 000 habitantes.

Del 1.o enero de 2019 al 31 de marzo de 2022 se notificaron 793 enfermedades huérfanas diferentes en Bogotá (aproximadamente un 35% de todas las enfermedades incluidas en la Resolución 023 de 2023). Las 20 patologías más frecuentes se identificaron en el 54,2% (5 636) de los reportes. La prevalencia total de las enfermedades huérfanas en Bogotá para el periodo del estudio fue de 13,4 por cada 10 000 habitantes; para el sexo femenino fue de 14,4 por cada 10 000 habitantes y para el sexo masculino fue de 12,2 por cada 10 000 habitantes. La enfermedad huérfana más frecuente fue la esclerosis múltiple con el 13,1% (1 363 casos). En la Tabla 3 se muestra la distribución de frecuencia de las 10 enfermedades huérfanas más prevalentes.

**Tabla 3:** Distribución y prevalencia de las 10 enfermedades huérfanas más frecuentes reportadas al Sivigila entre enero de 2019 y marzo de 2022 en Bogotá D. C.

Enfermedad	Mujeres		Hombres		Total	
	n (%)	Prevalencia	n (%)	Prevalencia	n (%)	Prevalencia
Esclerosis múltiple	950 (69,69)	23,53	413 (30,30)	11,16	1363 (13,1)	17,59
Esclerosis lateral amiotrófica	201 (47,7)	5,00	220 (52,25)	5,95	421 (4,04)	5,42
Síndrome de Guillain-Barre	155 (41,44)	3,88	219 (58,55)	5,90	374 (3,6)	4,79
Déficit congénito del factor VIII	63 (17,45)	1,53	298 (82,55)	8,01	361 (3,47)	4,67
Displasia broncopulmonar	126 (36,84)	3,10	216 (63,16)	5,80	342 (3,28)	4,40
Cirrosis biliar primaria	273 (89,8)	6,80	31 (10,2)	0,80	304 (2,92)	3,90
Enfermedad de Devic	202 (77,99)	5,01	57 (22,01)	1,55	259 (2,48)	3,37
Enfermedad de Von Willebrand	182 (70,81)	4,48	75 (29,18)	1,97	257 (2,47)	3,27
Esclerosis sistémica cutánea limitada	214 (90,3)	5,33	23 (9,7)	0,58	237 (2,27)	3,10
Hipertensión arterial pulmonar idiopática	164 (70,1)	4,03	70 (29,9)	1,90	234 (2,25)	3,06

Prevalencia multiplicada por coeficiente de 100 000 habitantes.

En la Tabla 4 se presenta el mismo análisis para las 5 más frecuentes de estas enfermedades por año, según grupo etario.

**Tabla 4:** Distribución y prevalencia de las 5 enfermedades huérfanas más frecuentes por grupos etarios reportadas al Sivigila entre enero de 2019 y marzo de 2022 en Bogotá D. C.

Grupo Etario	Enfermedad	Mujeres		Hombres		Total	
		n (%)	Prevalencia	n (%)	Prevalencia	n (%)	Prevalencia
0 - 5	Displacía broncopulmonar	85 (35,42)	29,40	155 (64,58)	51,40	240 (19,8)	40,90
	Microtia	24 (36,92)	8,30	41 (63,08)	13,60	65 (5,35)	11,10
	Gastrosquisis	17 (42,5)	5,90	23 (57,5)	7,60	40 (3,3)	6,80
	Déficit congénito de factor VIII	1 (2,7)	0,30	36 (97,3)	11,90	37 (3)	6,30
	Fibrosis quística	12 (41,93)	4,15	16 (58,06)	5,33	28 (2,55)	4,79
6 a 12	Displasia broncopulmonar	39 (41,05)	11,70	56 (58,95)	16,20	95 (7,1)	14,10
	Microtia	25 (37,14)	7,50	43 (62,86)	12,51	68 (5,23)	10,10
	Síndrome de Turner	51 (100)	15,39	0	0,00	52 (4,03)	7,70
	Déficit congénito de factor VIII	3 (6,38)	0,90	43 (93,62)	12,51	46 (3,5)	6,85
	Hiperplasia suprarrenal congénita	28 (63,64)	8,40	15 (36,36)	4,31	43 (3,3)	6,35
13 a 18	Déficit congénito de factor VIII	5 (11,76)	1,58	44 (88,23)	13,79	49 (5,6)	7,78
	Enfermedad de Von Willebrand	26 (72,97)	8,38	9 (27,03)	2,79	35 (4,1)	5,58
	Artritis juvenil idiopática de inicio sistémico	20 (60,6)	6,40	13 (39,4)	470	33 (3,64)	5,20
	Artritis relacionada con entesitis	7 (22,58)	2,2 0	22 (77,42)	6,88	29 (3,42)	4,58
	Poliartritis factor reumatoide positivo	23 (93,1)	7,41	2 (6,9)	0,60	25 (3,2)	3,97
19 a 24	Esclerosis múltiple	48 (61,54)	11,50	30 (38,46)	7,20	78(13,6)	9,40
	Enfermedad de Von Willebrand	30 (72,1)	7,16	11 (27,9)	2,66	41(7,5)	4,96
	Déficit congénito ctel factor VIII	4 (11, 43)	1,00	30 (88,57)	7,26	34 (6,12)	4,08
	Artritis juvenil idiopática de inicio sistémico	16 (59,26)	3,80	11 (40,74)	2,70	27 (4,7)	3,20
	Enfermedad de Devic	15 (85)	3,6 2	2 (15)	0,47	17 (3,5)	2,04
25 a 60	Esclerosis múltiple	774 (70)	36,60	328 (30)	17,13	1102 (25,77)	27,31
	Síndrome de Guillain-Barre	87 (42,31)	4,09	120 (57,69)	6,22	207 (4,74)	5,18
	Enfermedad de Devic	152 (76)	7,25	48 (24)	2,52	200 (4,56)	5,00
	Esclerosis lateral amiotrófica	83 (45,63)	3,90	95 (53,37)	5	178(4,06)	4,4
	Déficit congénito del factor VIII	46 (28,57)	2,20	116 (71,43)	6,3	162 (3,8)	4,2
>60	Esclerosis lateral amiotrófica	117 (48,8)	20,60	125 (51,2)	30,9	242 (12,47)	25,3
	Esclerosis múltiple	110 (72,48)	19,35	42 (27,52)	10	152 (7,49)	15,2
	Cirrosis biliar primaria	122 (90,37)	21,40	13 (9,63)	3,2	135 (6,8)	13,8
	Síndrome de Guillain-Barre	53 (40,15)	9,30	79 (59,85)	19,2	132 (6,63)	13,5
	Esclerosis sistémica cutánea limitada	91 (89,52)	15,97	11 (10,48)	2,7	102 (5,28)	10,7

Prevalencia multiplicada por coeficiente de 100 000 habitantes.

**Figura 3:**
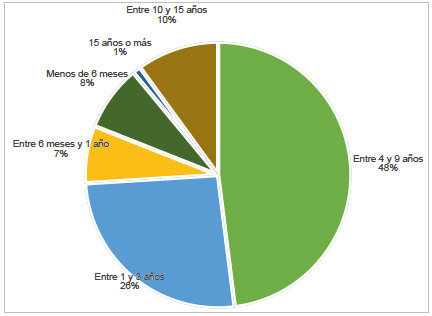
Distribución de la muestra en función de la demora diagnóstica

Un paciente con una enfermedad huérfana en Bogotá tarda, desde que inician sus síntomas hasta obtener un diagnóstico, 61,3 meses (desviación estándar de 101,9). En la Figura 3 se muestra la distribución de la población en función de la demora diagnóstica. El déficit congénito de factor VIII hace parte del grupo de las 10 enfermedades huérfanas más reportadas que en promedio más tardaron para la confirmación diagnóstica, con un promedio de 140,4 meses (DE 185,7). La Tabla 5 muestra el promedio y la mediana en meses del tiempo transcurrido entre el inicio de los síntomas y la fecha de confirmación diagnóstica de las 5 enfermedades huérfanas más frecuentes en función de la demora diagnóstica. Para dicho análisis se usaron 7 207 reportes.

**Tabla 5:** Promedio y mediana en meses del tiempo transcurrido entre el inicio de los síntomas hasta confirmación del diagnóstico de las 5 enfermedades huérfanas más frecuentes en función de la demora diagnóstica reportadas al Sivigila entre enero de 2019 y el marzo de 2022 en Bogotá D. C.

	Enfermedad	Promedio en meses	Mediana	Desviación estándar
15 años o más	Raquitismo hipofosfatémico familiar ligado al cromosoma X	207	145,4	212,3
	Malformación de Ebstein	208,3	55	287,8
	Ictiosis ligada a cromosoma X	216,3	214	96,6
	Síndrome de Marinesco-Sjogren	228	109,57	241,2
	Ictiosis lamelar	206,9	188	78,7
Entre 10 y 15 años	Déficit congénito de factor VI11	140,4	43,2	185,7
	Déficit congénito de factor IX	140,5	5,17	215,5
	Síndrome de Marfan	153,9	67,2	217
	Esclerosis tuberosa	120,4	47,87	165
	Angioedema hereditario	135,9	57,48	185,9
Entre 4 y 9 años	Esclerosis múltiple	69,9	24,8	92,3
	Esclerosis sistémica cutánea limitada	67,2	44,7	73,4
	Esclerosis sistémica cutánea difusa	66,8	45,1	70,6
	Hipertensión arterial pulmonar idiopática	51,5	25,7	72,8
	Enfermedad de Von Willebrand	102,6	44,9	136,6
Entre 1 y 3 años	Esclerosis lateral amiotrófica	22,1	12,5	29,7
	Enfermedad de Devic	22	6,4	34,1
	Cirrosis biliar primaria	40,5	18,2	56,5
	Enfermedad de Crohn	45,3	19,1	64,3
	Fibrosis pulmonar idiopática	26,8	23,2	26,4
Entre 1 año y 6 meses	Displasia broncopulmonar	10,3	4,7	18,5
	Síndrome de intestino corto	7,8	4,1	11,4
	Síndrome hemolítico urémico atípico	10,6	0,13	32,4
	Aplasia medular idiopática	8	2,1	15
	Síndrome de West	7,7	0	15,2
Menos de 6 meses	Síndrome de Guillain-Barre	2,5	0,13	13,4
	Gastrosquisis	0,4	0	1,5
	Porfiria aguda intermitente	5,7	1,7	7,9
	Coartación atípica de aorta	0,3	0	0,9
	Plagiocefalia aislada	4,9	4,08	5

## DISCUSIÓN

Se estima que la prevalencia de las enfermedades huérfanas oscila entre el 6 y el 8%. Este valor se estimó teniendo en cuenta 5 000 a 8 000 enfermedades 13, mientras que en la Resolución 023 del 2023 se incluyeron 2 247. Por ende, en nuestro estudio se observó un subregistro, ya que la prevalencia de enfermedades huérfanas fue de 13,4 por cada 10 000 habitantes (0,13%). Esto implica que podría haber, aproximadamente, 500 000 personas con una enfermedad huérfana en Bogotá que no han sido notificadas. Un factor asociado al bajo número de casos notificados al Sivigila es el desconocimiento de la obligatoriedad de la notificación de estas enfermedades. La reducción del 42% de la notificación en Bogotá de 2019 a 2020 se debió a la disminución en el número de consultas de atención primaria y urgencias por la pandemia covid-19, ya fuese por miedo a exposición en las instituciones de salud, o por limitaciones de movilidad por el confinamiento impuesto por el Gobierno nacional 14,15.

Más de la mitad de los casos de enfermedades huérfanas corresponde a mujeres. Esto podría explicarse porque una importante cantidad de enfermedades huérfanas son inmunológicas y se ha establecido que las enfermedades autoinmunes tienen una mayor prevalencia en mujeres 16; además, las mujeres utilizan más los servicios de salud que los hombres 17. Con relación a la distribución por edad, se esperaría que la notificación predominara en la edad pediátrica, pues el 50% de las enfermedades huérfanas aparece en este periodo de la vida, sin embargo, en nuestro análisis la notificación predomina en la edad adulta, lo que se explica por la excesiva mortalidad de muchas de estas enfermedades; se estima que el 30% de estos pacientes fallece antes de los 5 años, y el 50% antes de los 30 años 18.

El grupo de edad con más casos fue el de 0 a 12 años, en el que más de la mitad de los reportes corresponde a hombres, lo que concuerda con lo observado en otros estudios, según los cuales los hombres tienen mayores posibilidades de ser prematuros y padecer afecciones derivadas como lo es la displacia broncopulmonar 19,20, la enfermedad huérfana más reportada en dicho grupo de edad en nuestra población. Si bien el diagnóstico de displasia broncopulmonar se hace en el periodo neonatal, el hecho de que se reporten diagnósticos hasta los 12 años puede indicar notificación tardía por desconocimiento de la obligatoriedad de reporte o un diagnóstico retrospectivo por deterioro funcional pulmonar secundario tardío.

La gran mayoría de los casos pertenece al régimen contributivo. Esto se explica porque para el 2019 más del 80% de la población en Bogotá se encuentra afiliada a este régimen y aproximadamente el 15% al subsidiado, cifras que no han presentado mayores variaciones hasta el 2022 21. Además, las personas pertenecientes al régimen subsidiado suelen tener inestabilidad laboral y financiera, lo que limita el acceso a los servicios de salud y genera una menor oportunidad de diagnóstico 22.

Con respecto a la residencia de los pacientes con enfermedades huérfanas discriminada por localidad, se evidencia que se concentra en Suba, Kennedy y Engativá, localidades con mayor densidad poblacional. Sin embargo, la representación de los pacientes por localidad no tiene un patrón predecible y lo que determina donde serán atendidos es la red de IPS que tenga la EAPB a la que se encuentren asegurados.

El presente estudio, similar a lo reportado por Mazzucato et al. mostró que las patologías con mayor prevalencia en los adultos son las que afectan de manera importante la locomoción y la visión, que conllevan discapacidad y limitaciones para el ámbito laboral, lo cual contribuye a que estas patologías se diagnostiquen con mayor frecuencia 23.

En el actual estudio, la esclerosis múltiple fue la enfermedad más notificada, con una prevalencia de 17,59 casos por cada 100 000 personas. En el mundo, la esclerosis múltiple tiene una prevalencia de 35,9 casos por cada 100 000 personas (363 casos por cada 100 000 personas en Estados Unidos; 108 por cada 100 000 personas en Europa) 24,25. En Latinoamérica se registra una heterogeneidad en los resultados, los cuales varían desde 5,05 casos por cada 100 000 personas en Quito, 15 por cada 100 000 personas en San Pablo, hasta 38,2 por cada 100 000 personas en Buenos Aires. La diferencia en la prevalencia de Europa y Estados Unidos con los países Latinoamericanos se explica posiblemente porque la población caucásica tiene un riesgo más alto que los no caucásicos de tener esclerosis múltiple, incluso puede que esta sea la razón de que la prevalencia en Buenos Aires sea mayor a la reportada en Bogotá y Quito, pues hay mayor población de origen caucásico en las regiones del sur de Latinoamérica que en Colombia y las regiones del norte donde predominan los mestizos 26.

En cuanto a la esclerosis lateral amiotrófica (ELA), la prevalencia mundial es de aproximadamente 6 casos por cada 100 000 personas (5,2 casos por cada 100 000 personas en Estados Unidos; 6,22 casos por cada 100 000 personas en Europa) 27. En Latinoamérica la preva-lencia varia de 0,9 por cada 100 000 personas en Brasil, 1,9 por cada 100 000 personas en Uruguay, hasta 8,86 por cada 100 000 personas en Argentina 28. La prevalencia calculada a partir de la notificación encontrada en nuestro estudio fue de 5,42 por cada 100 000 personas, valor cercano a la prevalencia mundial. Dado que Bogotá tiene una población mayoritariamente mestiza, nuestros hallazgos contrastan con la de otros autores quienes encontraron una mayor prevalencia de ELA en caucásicos frente a no caucásicos 29, por ello se propone que los estilos vida pueden estar contribuyendo a la aparición de ELA en nuestra población 30.

Se ha reportado que la prevalencia del déficit congénito de factor VIII o hemofilia A es de 12,8 casos por cada 100 000 hombres en países de altos ingresos y de 6,6 casos por cada 100 000 hombres en países de bajos ingresos 31. En nuestro estudio la prevalencia calculada a partir de la notificación fue de 8,01 casos por cada 100 000 hombres. La enfermedad de Von Willebrand tiene una prevalencia entre 0,01 y 1% en la población mundial 32; en el presente estudio fue de 0,003%. No se ha descrito una predilección étnica de dichas patologías 31,33, por lo cual las bajas prevalencias observadas en nuestro estudio de la hemofilia A y de la enfermedad de Von Willebrand podrían deberse a un diagnóstico inexacto o tardío por dificultades en el acceso a pruebas diagnósticas, imposibilidad de consulta con equipo médico especializado y falta de disponibilidad de tratamientos pertinentes, lo que en su conjunto puede llevar a una muerte prematura. Esta dificultad en el acceso a recursos tecnológicos o de personal entrenado también podría contribuir a la baja prevalencia observada en las otras enfermedades huérfanas.

Otro factor que puede contribuir a la baja prevalencia de las enfermedades huérfanas observada en el presente estudio, como se ha mencionado, es la subnotificación del evento, asociado al desconocimiento del personal de salud en torno a los eventos de reporte obligatorio al Sivigila, por lo que sí se están diagnosticando dichas enfermedades en nuestro medio, pero no se notifica por desconocimiento del personal de salud. Si se toma en cuenta la enfermedad huérfana más notificada en este estudio, que es la esclerosis múltiple, la prevalencia descrita en la literatura es de 35,9 casos por cada 100 000 24; con esa prevalencia en Colombia deberíamos tener alrededor de 18 000 casos y hasta el final del 2022 se habían notificado solo 4 264 en todo el país 34.

El retraso diagnóstico de las enfermedades huérfanas es algo ya documentado en otras poblaciones. Un reciente estudio español, en el que se utilizaron datos del Registro de Pacientes de Enfermedades Raras del Instituto de Salud Carlos III, estimó que para 2021 el 20,9% de los pacientes con una enfermedad huérfana tuvo una demora diagnóstica de 10 años o más, el 19% entre uno y 3 años, el 16,7% entre 4 y 9 años y el 43,6% menos de un año 35. En nuestro estudio, el 11% de los pacientes con una enfermedad huérfana tuvo una demora diagnóstica de diez años, en un 26% entre uno y 3 años, el 48% entre 4 y 9 años y un 15% menos de un año. Las diferencias de nuestros resultados con los de España pueden deberse a que dicho país lleva más años de experiencia en la implementación de programas para la investigación y el cuidado de pacientes con enfermedades raras, pues desde 2003 el Gobierno español, por medio del Instituto de Investigación de Enfermedades Raras, viene fomentando la investigación clínica y básica en la atención de la salud en enfermedades raras, y en 2005 se estableció un registro nacional de enfermedades raras 36). Mientras tanto, en Colombia las enfermedades huérfanas se reconocieron como un problema de especial interés en salud pública con la Ley 1392 de 2010, y no fue hasta 2016 que se estableció un registro nacional de enfermedades huérfanas mediante el Sivigila 4,37.

En el presente estudio un paciente con una enfermedad huérfana tarda en promedio 5 años en obtener un diagnóstico. Bogotá es de las ciudades que cuenta con una mayor cantidad de clínicas y hospitales de alta complejidad con disponibilidad de técnicas diagnósticas y talento humano especializado. Se puede inferir que, si en el análisis se incluyen zonas como Amazonas, Guainía, San Andrés, Chocó, las cuales se han relacionado tradicionalmente con pobreza y por ende con limitaciones en el acceso a los servicios de salud, el valor promedio del tiempo de retraso diagnóstico para Colombia aumentaría.

Los resultados de este estudio indican que hay un claro retraso en el diagnóstico de las enfermedades huérfanas en Bogotá. Esto, debido a que como señalan Berrocal-Acedo et al., cuando se produce un primer contacto con la atención sanitaria, habitualmente no se sospecha de una enfermedad huérfana, sino que primero se descartan las enfermedades más comunes y se realizan pruebas diagnósticas que, en la mayoría de los casos, no ofrecen resultados esclarecedores. Tras esto, comienza a derivarse a especialistas, y por la heterogeneidad que manifiestan las enfermedades huérfanas en su historia natural, su forma de presentación clínica diversa e inespecífica, sumado a su baja prevalencia, hacen de esta situación una odisea diagnóstica 38. Así, dicho retraso diagnóstico tiene graves consecuencias sobre el curso natural de la enfermedad, pues como mostró el estudio ENSERio (Estudio de necesidades socio sanitarias de afectados por enfermedades raras) realizado en España, en el 31,26% de las personas con una enfermedad huérfana, el retraso en el diagnóstico produjo un agravamiento de su enfermedad o de sus síntomas, el 29,37% no recibió ningún apoyo ni tratamiento y el 17,90% fue sometido a tratamientos inadecuados 39 ♠
